# Mental workload accumulation effect of mobile phone distraction in L2 autopilot mode

**DOI:** 10.1038/s41598-022-17419-1

**Published:** 2022-10-07

**Authors:** Hongfei Zhao, Jinfei Ma, Yijing Zhang, Ruosong Chang

**Affiliations:** School of Psychology, Liaoning Normal University, Dalian, 116029 China

**Keywords:** Human behaviour, Psychology, Risk factors

## Abstract

As automated vehicles become more common, there is a need for precise measurement and definition of when and in what ways a driver can use a mobile phone in L2 autonomous driving mode, for how long it can be used, the complexity of the call content, and the accumulated mental workload. This study uses a 2 (driving mode) × 2 (call content complexity) × 6 (driving stage) three-factor mixed experimental design to investigate the effect of these factors on the driver's mental workload by measuring the driver's performance on Detection response tasks, pupil diameter, and EEG components in various brain regions in the alpha band. The results showed that drivers' mental workload levels converge between manual and automatic driving modes as the duration of driving increases, regardless of the level of complexity of the mobile phone conversation. This suggests that mobile phone conversations can also disrupt the driver's cognitive resource balance in L2 automatic driving mode, as it increases mental workload while also impairing the normal functioning of brain functions such as cognitive control, problem solving, and judgment, thereby compromising driving safety.

## Introduction

The use of mobile phones while driving is a widespread phenomenon, with various countries banning the use of mobile phones for talking while driving and drivers and the public becoming aware of the negative effects of mobile phone use while driving, yet the proportion of drivers using mobile phones while driving is still increasing every year^1^. Mobile phone conversations contribute to reduced driving performance and increased crash probability in a number of ways (e.g. increased cognitive load on drivers, longer reaction times to events, etc.)^2–4^.

At the same time, despite the popularity of autonomous driving vehicles, there are no clear legal regulations in these countries regarding whether the use of mobile phones is allowed while driving smart vehicles. Current laws and regulations in the field of autonomous driving only govern the manufacturing technology of autonomous driving vehicles, the public road testing of autonomous driving vehicles, and the systems of ethical reasoning when conducting autonomous driving. For example, safety assessment standards for autonomous driving vehicles are limited within each state in the US, China has clear requirements for road specifications for testing autonomous driving vehicle technology, and the UK and Australia require drivers to be ready to take over and control the vehicle^5^. The American Society of Automotive Engineers and the National Highway Traffic Safety Administration have defined six levels of autonomous vehicles from L0 to L5, based on the level of vehicle automation^6^. The most widespread vehicles on the market today are those with L2 level of driving, also known as 'Partly automated driving', which combines both longitudinal and lateral control of the vehicle and is represented by the vehicle's adaptive cruise control (ACC) and lane center assist. L2 autonomous driving systems require the driver to continuously monitor road hazards and be ready to take over the vehicle at all times.

Rudin-Brown et al.^7^ showed that when drivers used adaptive cruise control (ACC), they were more likely to engage in secondary tasks (e.g., making phone calls, using the radio, etc.) and they took longer to detect hazards than when they did not use the ACC system. Llaneras et al.^8^ found that when drivers used L2 automated driving systems while engaged in secondary tasks, the duration of the driver's vision away from the road ahead increased. Research by Gasper et al.^9^ also confirms that with the use of L2 automated driving systems, drivers will develop longer visual disengagement, spend more time with their eyes on the in-vehicle dashboard and on in-vehicle aids such as operating screens, and require longer times to take over the automated vehicle compared to traditional manual driving, with increased reaction time. These studies suggest that driver engagement in distracting tasks during L2 level driving can have a range of negative effects on driver performance. Therefore, there is a need to precisely measure and define when and in what manner drivers can use mobile phones in L2 automated driving mode (The automated driving mode discussed below are all L2-level autonomous driving systems), how long they can continue to do so, as well as the complexity of the calls and the accumulated mental workload, in order to provide a theoretical basis for the development of a system to regulate and classify mobile phone use in L2 automated driving mode.

### Indicators for testing the mental workload of drivers

The main methods of monitoring drivers’ mental workload include subjective assessment, detection response task (DRT), eye tracking, and EEG measurements. A large number of studies on cognitive load have employed the ISO standardized Detection Response Task, DRT^10^. DRT requires participants to press a button as fast as possible upon detection of a visual or tactile stimulus presented at irregular intervals of 3–5 s. Results from DRT experiments consistently showed that, regardless of stimulus type, cognitive load resulted in a significant increase in reaction time to DRT stimuli compared to baseline (no-task) conditions^11,12^. Other researchers have used pupil diameter to measure driver mental workload^13^, compared with the primary driving task, driver’s pupil diameter increased when they performed a secondary distraction task simultaneously^14^.

Changes in drivers' electrical brain activity (Electroencephalo-graph, EEG) can also be a useful indicator of their distracted state and mental workload, and electroencephalographic component (EEG) measurements are the most direct way means used to detect driving distractions^15,16^. Brookhuis et al.^17^ used a driving simulator to test drivers' driving performance in different road environments and recorded their ECG and EEG signals, and found that the alpha band of the EEG signal correlated well with drivers' mental workload, with a significantly lower alpha wave band power spectrum at higher mental workload^18^. A study by Almahasneh et al.^19^ examined the effects of different cognitive tasks (mathematical calculations and decision problems) on driver cognitive state. It was found that the area most affected during distracted driving was the right frontal cortex region, and that activation of the right frontal cortex region was effective in examining the driver's cognitive distraction state. Additionally, changes in the right frontal alpha band may also be a better indicator, which needs to be further validated in future studies^20^.

At the same time, monitoring the driver's attention-related brain resources remains a challenge for researchers in the field of cognitive brain research and human–computer interaction^20^. Frontal regions are known to be involved in impulse control, judgement, language production, working memory, motor function, and problem solving.^21^Activation of frontal areas is induced by the performance of mental tasks. In turn, power changes in frontal areas represent the degree of activation of intrinsic neurons when individuals allocate their attention to different task stimuli^22^. The prefrontal cortex in frontal areas has also been thought to play an important role in cognitive control, i.e. the coordination of thoughts and actions according to internal goals^23^. Therefore, the detection of EEG changes during a driver's mobile phone call is expected to further uncover the hazards and effects on various brain regions.

Based on the above literature review, this study used a multimodal detection method to simultaneously measure drivers' detection response task performance, pupil diameter and EEG components across brain regions in the alpha band to comprehensively assess drivers' mental workload in both automated and manual driving conditions. This will provide guidance suggestions for the development of an EEG-based distraction detection and intervention system for drivers' mobile phones.

### Effects of mobile phone distraction on driver mental workload in autonomous driving mode

Many studies have shown that drivers tend to use their mobile phones during the use of automated driving systems^24^. A study by Noble et al.^25^ found that drivers frequently engaged in high-risk secondary tasks (e.g., browsing their mobile phones, dialing numbers with their phones in hand, using their mobile phones for location, etc.) in L2-level autonomous driving mode, thereby prolonging the time their eyes are off the road. Banks et al.^26^ found that some drivers even took their hands off the steering wheel for up to 11 s while using their mobile phones in L2 autonomous driving mode. These studies all suggest that drivers are prone to mobile phone distractions during automated driving.

It has also been shown that drivers attempt to increase the mental workload in monotonous environments to control their increasing levels of passive fatigue. Young and Stanton, in a summary of the extensive literature, defined mental workload as the amount of attentional resources people give to meet objective and subjective performance criteria, which is related to task demands, external support, and the individual's experience^21,27,28^. For example, Neubauer's^29^ study showed that using a mobile phone and doing something unrelated to the driving task while in the car was effective in maintaining driver engagement during autonomous driving. A study by Atchley^2^ found that strategic language tasks improved driver performance and alertness during fatigue. This suggests that the additional load imposed by mobile phone conversations may be beneficial to drivers in reducing passive fatigue and helping to maintain their alertness.

However, when drivers are driving for long periods of time and are already actively fatigued, short-term strategies to increase task load and arousal levels are unlikely to have much benefit in terms of active fatigue relief. In fact, when fatigued drivers engage in mobile phone conversations, these conversations and fatigue superimpose to crowd attentional channels and the driver's mental workload increases, resulting in a cumulative effect^30^). Saxby et al.^4^ showed that mobile phone conversations do not counteract fatigue induced by autonomous driving and that mobile phone conversations in a state of passive fatigue may further impair driver driving performance. However, this study only measured driver fatigue from a subjective rating perspective and did not differentiate driver fatigue levels by stage, nor did it differentiate the complexity of the content of mobile phone calls, and therefore could not provide targeted guidance on policy recommendations for mobile phone use regulations under autonomous driving conditions.

### Impact of mobile phone call task complexity on driver mental workload

Research has found that the degree of difficulty (task complexity) or emotionality of a driver making a mobile phone call while driving can affect the driver's cognitive demands, which may distract the driver from the driving task^31^.

Current research on the effects of mobile phone conversation task complexity on drivers' mental workload can be broadly divided paradigmatically into two categories: computational reasoning-type tasks, represented by similar tasks such as logical reasoning and mathematical calculations, and naturalistic contextual or emotional mobile phone conversation tasks. In a study by Shinar et al.^32^, it was found that drivers performing logical reasoning tasks reduced driving performance to a greater extent than engaging in conversations involving emotional relevance. The advantages of this type of task are that the experiment is well controlled, the complexity is clearly quantified, and the experiment is more effective. However, the disadvantage is that they lack ecological validity and are difficult to generalize to everyday contexts.

Another category of naturalistic contextual or emotional phone conversation task is more reductive to real driver phone conversation content. A study by Al-Tarawneh et al.^33^ found that a recall-type phone conversation task (representing complex call content) had a much higher response latency effect on visual targets than having a simpler everyday conversation. Although the ecological validity of this type of task is high, subsequent studies are difficult to replicate, for example, Rakauskas et al.^34^ used a call task in a naturalistic context to investigate the relationship between call difficulty and driver distraction. The results showed that although mobile phone use reduced driving performance, the level of call complexity did not have a significant effect on average speed, driving performance, or mental workload. The reason for the inconsistency of this study's results with other similar studies may be that conversations in natural contexts require less cognitive load than the verbal reasoning and mathematical tasks used in other studies, and therefore the effect of increased complexity of call content is less sensitive to driving performance. All of the above studies suggest that the effect of naturalistic contexts or emotional mobile phone call content on driving performance is limited and dependent on the experimenter's control over the complexity of the call task. Based on these considerations, the present study selected call content related to logical reasoning to examine the effects of mobile phone distractions on driver performance on mental workload and vigilance detection tasks.

In addition to the complex of mobile phone call content, another difficulty in the development of laws and regulations regarding the use of mobile phones for autonomous driving is the determination of call duration. The question of how long a call is beneficial for the mitigation of passive fatigue in autonomous driving mode is also a focus that this study explores. A recent study shows that the first 40 min of a driving task under monotonic autonomous driving conditions is a critical period for passive fatigue to develop^35^, and we thus envisage whether the driver underload problem would be alleviated if mobile phone call content is imposed during the first 40 min of a monotonic autonomous driving task, and as the driving duration increases, we ask whether the amount of load caused by mobile phone calls during autonomous driving differs from that of manual driving. This study thus uses a 2-(driving mode: automatic driving group, manual driving group)*2 (call content complexity: simple call content group, complex call content group)*6 (driving stage: 6 stages) three-factor mixed experimental design to examine the effects of driving mode, call content difficulty, and driving phase on driver mental workload.

The following hypotheses were proposed:

During the initial phase of driving (within 40 min), the EEG alpha wave power values were higher in the autopilot simple talk content group than in the manual simple talk content group and tended to increase. When the driving time was 60 min, the EEG alpha power values of mobile phone calls in the autopilot mode did not differ from those of the manual driving group.

During the initial phase of driving (within 40 min), when drivers were making mobile phone calls, the detection response task reaction time was slower in the manual driving group than in the automatic driving group, and the detection response task correctness rate was lower in the manual driving group than in the automatic driving. As the driving time increased (around 60 min), the detection response time became slower and the correctness rate decreased in the automatic driving group, and their task performance converged with that of the manual driving group.

During the initial phase of driving (within 40 min), the pupil diameter was smaller in the autopilot simple talk content group than in the manual driving group. When driving for 60 min, there was no significant difference between the pupil diameter of the autopilot group and the manual driving group.

## Methods

### Participants

Recruiting 58 college student novice drivers with driving licenses in Dalian, 29 male and 29 female. The age range was 20–30 years (*M* = 22.03, *SD* = 2.08), the driving experience range was 1–5 years *(M* = 1.85, *SD* = 1.56) and the mileage range was 1–1000 km (*M* = 329.41, *SD* = 402.105). Eyeglass wearers were also questioned and were asked to participate in the experiment with both right and left eye prescriptions controlled to less than 200 degrees and without problems such as astigmatism. The subjects were randomly assigned to 15 subjects in the automatic driving mode simple talk group (AS), 15 in the automatic driving mode complex talk group (AC), 14 in the manual driving mode simple talk group (MS) and 14 in the manual driving mode complex talk group (MC). The EEG data of 7 subjects were excluded due to a large number of artefacts caused by large head movements and the EEG data were not collected in full at some electrode sites. The EEG data was finally valid for 51 participants, the valid data for driving performance was 58, and the valid data for pupil diameter was 58. Subjects were asked to refrain from drinking alcoholic or caffeinated beverages 24 h prior to the experiment, to get enough sleep the day before the experiment, and were given a reward at the end of the experiment. This study was approved by the Ethics Committee of Liaoning Normal University and was performed in accordance with the approved guidelines and the Declaration of Helsinki. All the participants provided written informed consent before participating.

### Experimental design

The experimental design was a 2 (driving mode: automatic driving group, manual driving group)*2 (call content complexity: simple call content group, complex call content group)*6 (driving stages: 6 stages) three-factor mixed experimental design. The driving stages were evenly divided according to the driving duration of 1 h into 6 stage, with 0–10 min as the first stage, 10–20 min as the second stage, 20–30 min as the third stage, 30–40 min as the fourth stage, 40–50 min as the fifth stage, and 50–60 min as the sixth stage. The driving mode and call content complexity are between-subject factors, the driving stage is a within-subject factor, and the dependent variable is the driver's mentao workload level, as indicated by the power of each brain region in the EEG *alpha* wave, the duration of the detection response task, the accuracy of the detection response task, and the pupil diameter size.

### Experimental materials

#### Mobile phone call task design

The content of the mobile phone call task is designed to distract the driver sufficiently to cause distraction and increase the driver's workload. Mobile phone call tasks are designed for two levels of difficulty: simple and complex, and Rakauskas et al.^34^ study suggests that the difficulty of mobile phone call content is differentiated by the level of cognitive load on the driver. The naturalistic nature of the conversation involving driver memory and recollection is considered simple, while arithmetic problems involving logical reasoning, calculation, or verbal confusion are considered difficult.

In the talk task, participants were asked to provide appropriate answers after listening to the complete question. The questions in the simple talk task were designed based on Burns’ et al.^36^ talk task. The questions asked in the simple talk task were conversational in nature in a natural context, e.g., "What is your favorite color?". The simple call task was ten questions. The complex call task, on the other hand, was designed based on the call task of Peng et al.^37^. Some arithmetic questions or some verbal confusion (requiring participants to reason logically) questions were presented to participants, e.g. "If Kris is younger than Albert and Albert is younger than Sam, then who is the youngest?" The complex call task was also a ten-question task.

The call task was played back to the participant in the form of a hands-free mobile phone (JBL wireless Bluetooth audio) during the experiment, and the subjects were asked to answer the call content question as soon as they heard it. The duration of the experiment was 60 min, divided equally into six phases, each phase being three minutes in length of the mobile phone call.

### Driving setting

**Figure 1:**
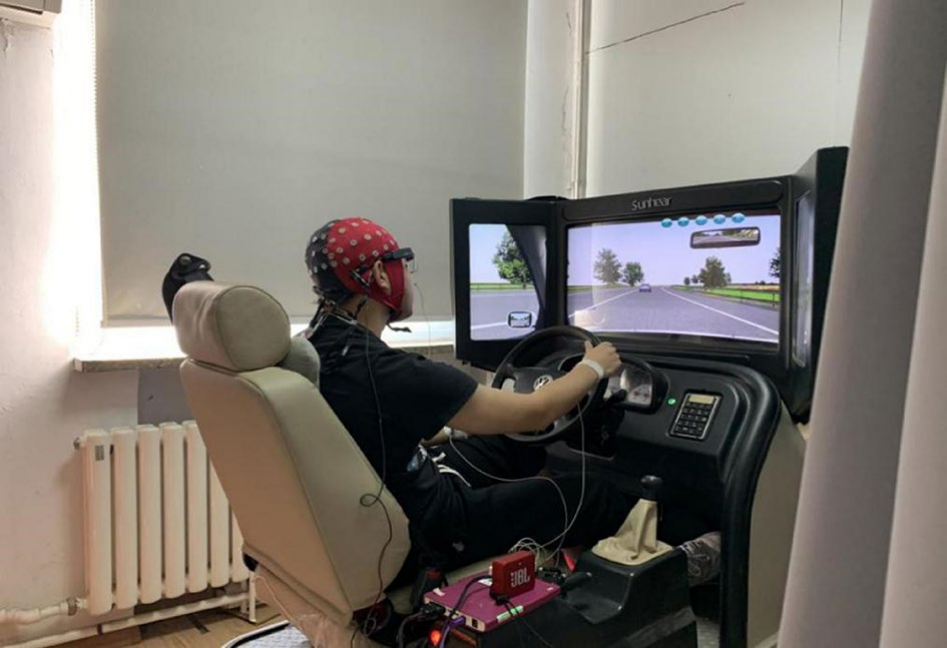
Driving tasks and simulated driving scenarios.

This experiment used the Xuan Ai QJ-3A1 (small) driving simulator with constituent components such as a seat belt, steering wheel, instrument panel, transmission lever, parking brake operating lever, brake pedal, and accelerator pedal, which accurately replicates the interior of a small motor vehicle cab. See Fig. 1. This study kept the cognitive load low during driving with few stimuli and low driving task difficulty. A daytime, sunny urban roadway was used as the simulated driving scenario. In the manual driving mode, participants were asked to follow the vehicle in front of them normally, travel at a speed of no more than 120 km/h and maintain a safe distance (no less than 100 m) from the vehicle in front of them at all times while driving. The participants were also asked to perform a detection response task presented randomly by the screens on both sides of the driving simulator, i.e. to brake in response to a picture of a pedestrian appearing on the screen, with a randomized presentation time of between 60 ± 40 s.

In automatic driving mode, the driver was not required to steer or apply the brakes. Participants were asked to monitor road conditions at all times and needed to step on the brakes to perform detection response tasks, the other experimental conditions were the same as in manual driving mode.

### Pupil diameter measurement

This experiment used a head-mounted Tobii Pro Glasses II eye-tracking system (Tobii Pro Glasses II, Sweden) to record eye movement data, which allowed free head movement. The oculomotor was sampled at a frequency of 50 Hz and had an accuracy of 0.5°. The subjects' eye movement data was collected and analyzed using Tobii Studio 3.0. Pupil diameter data was mainly collected from the subjects.

### Experimental procedure

#### Experimental preparation stage

To briefly introduce the procedure, the subject was given an EEG and eye-tracking device and asked to fill in basic information, including age, gender, driving age, and education level. To ensure that the subjects were familiar with the procedure, they were first provided with verbal instructions to practice using the simulator for 3–5 min, which included using the simulator equipment, giving braking responses to random event stimuli on the screens on both sides of the simulator, and answering talking questions played on the audio to ensure that the subjects learned how to use it.

#### Application phase

Subjects performed a 60-min driving task and completed a call task of the corresponding group difficulty based on the assigned group. During the experiment, the driver was required to complete a detection response task (i.e. to brake in response to a picture of a pedestrian appearing on the screen, with a randomized presentation time of between 60 ± 40 s) in which 50 bursts appear randomly on both sides of the driving simulator screen, with each burst appearing at a random location and at random intervals to avoid expectation effects on the subject. The driver's eye movements and EEG data were also recorded.

At the end of the formal experiment a fee was given to the subject to verbally ask about the problems encountered in the driving simulation and the psychological situation, and to thank the subject for participating.

### EEG data acquisition

The experiments were conducted using a 64-lead EEG instrument to acquire EEG data in real time, using a sampling rate of 2000 Hz to amplify and digitize the signals. The international 10–20 electrode system was used to arrange the electrode positions, with the CPz electrode as the reference electrode and the AFz electrode as the ground electrode. The resistance of all electrodes was less than 10KΩ.

### EEG data pre-processing

According to the principle of resting EEG data preprocessing, after filtering the continuous EEG data between 0.5 and 30 Hz, the EEG data of each participant during the simulated driving process were divided into six parts, corresponding to stage 1–6, respectively. Each phase lasted about 10 min, and the sampling rate was reduced to 250 Hz. EEG data were referenced to the average of both mastoids (M1, M2). The Independent Component Analysis (ICA) algorithm was used to correct the part of the data contaminated by eye movement or electromyography (EMG) data or by any other non-physiological diseases.

### Power computation

For each participant, the pre-processed continuous EEG data were segmented into dozens of epochs, with epoch length of 2000 ms. Then the 61-channel segmented epochs were transformed to the frequency domain based on Fast Fourier transforms (FFTs) using a Hamming window with a 50% overlap, yielding FFTs ranging from 0.5 to 30 Hz with a frequency resolution of 0.5 Hz. The power spectrum of each frequency point was averaged over the epochs. Single-subject EEG spectra were averaged across subjects in each group in order to obtain group-level EEG spectra.

According to Brookhuis and Waard et al. (2014), the alpha band of the EEG signal was found to correlate well with the mental load of the driver. Accordingly, the EEG power in the alpha (8–13 Hz) band was calculated and the workload in five specific brain regions was examined and explored according to the electrode positions corresponding to different brain regions: frontal, F3, Fz, F4; temporal, T7, T8; parietal, P3, Pz, P4; occipital, O1, Oz, O2; and prefrontal, Fp1, Fpz, Fp2.

## Results

### Results of the detection response task analysis

Repeated-measures ANOVA tests were conducted with driving mode and call content complexity as between-subject variables, measurement phase as within-subject variables, reaction time and accuracy apart as the dependent variable, with Greenhouse–Geisser correction for p-values that did not satisfy the sphericity hypothesis variable. See Table 1 for the significance of factors.

**Table 1:** A summary table of the ANOVA significance of DRT (*p < 0.05).

Variables	Reaction time of DRT	DRT accuracy
Stage	0.567	0.104
Driving mode	0.582	0.328
Complexity	0.884	0.903
Driving mode*complexity	0.843	0.390
Stage*driving mode	0.008*	0.049*
Stage*complexity	0.890	0.433
Stage*driving mode*complexity	0.527	0.577

#### Results of the analysis of the reaction time

Results indicated a significant interaction between stage and driving mode, *F*(1, 54) = 7.598, *p* = 0.008, *ηp*^*2*^ = 0.123. Simple effect tests demonstrated that different driving modes appeared to differ significantly at stage 1, *p* = 0.000.

In the manual driving mode, regardless of the complexity of the call content, the driver's response time in the first stage is significantly different from that in the fourth stage (*p* = 0.007), the stage 1 and the stage 5 were significant (*p* = 0.010), and the stage 3 and the stage 4 were significant (*p* = 0.046).

In the autonomous driving mode, regardless of the complexity of the call content, the driver's reaction time between stages is not significant.

**Figure 2:**
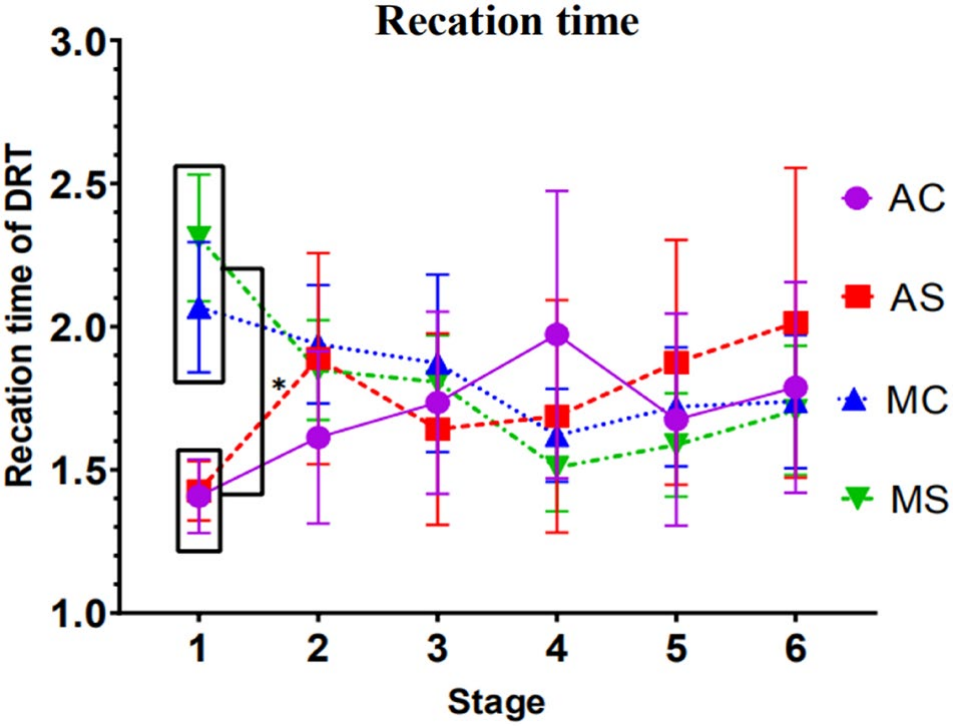
Trend in response time for detection response tasks (Note: Vertical bars represent standard errors, *p < 0.05).

Apart from this, no other factors or interactions reached significance. See Fig. 2 for detailed trends.

#### Results of the analysis of the accuracy

Results showed a significant interaction between stage and driving mode, *F*(1, 54) = 4.070, *p* = 0.049, *ηp*^*2*^ = 0.070. Simple effect tests demonstrated that different driving modes appeared to differ significantly at stage 1, *p* = 0.003.

In the manual driving mode, regardless of the complexity of the call content, the driver's accuracy in the first stage is significantly different from the second stage (*p* = 0.003), the stage 1 and the stage 4 were significant (*p* = 0.000), and the stage 1 and the stage 5 were significant (*p* = 0.003), the stage 1 and the stage 6 were significant (*p* = 0.020), the stage 3 and the stage 4 were significant (*p* = 0.020).

In the autonomous driving mode, regardless of the complexity of the call content, the driver's accuracy between stages is not significant.

**Figure 3:**
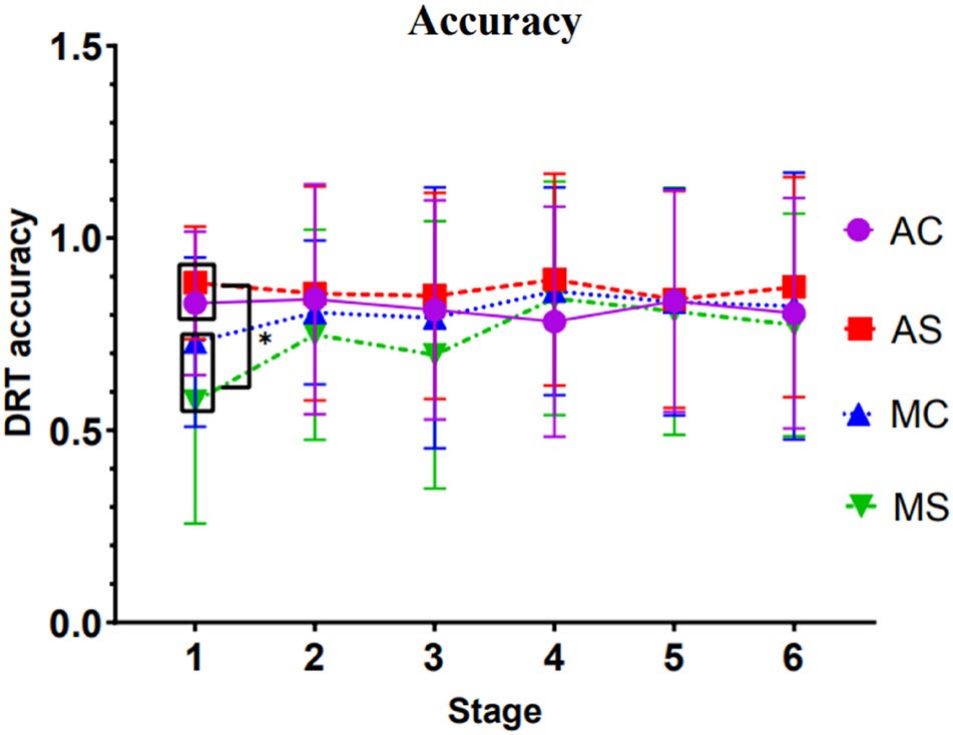
Trends in correct detection response task rates (Note: Vertical bars represent standard errors, *p < 0.05).

Apart from this, no other factors or interactions reached significance. See Fig. 3 for detailed trends.

### Results of pupil diameter analysis

Repeated measures ANOVA tests were conducted with driving mode and call content complexity as between-subject variables, measurement phase as within-subject variables, and pupil diameter as the dependent variable, with Greenhouse–Geisser correction for p-values that did not satisfy the spherical hypothesis variable. See Table 2 for the significance of factors.

**Table 2:** A summary table of the ANOVA significance of pupil diameter.

Variables	Pupil diameter of left eye	Pupil diameter of right eye
Stage	0.002*	0.005*
Driving mode	0.120	0.049*
Complexity	0.743	0.849
Driving mode*complexity	0.241	0.565
Stage*driving mode	0.005*	0.008*
Stage*complexity	0.130	0.126
Stage*driving mode*complexity	0.344	0.025*

#### Results of left eye pupil diameter analysis

Results showed a significant interaction between stage and driving mode *F*(1, 54) = 8.571, *p* = 0.005, *ηp*^*2*^ = 0.137. Simple effect tests demonstrated a significant difference between driving modes at stage 1, *p* = 0.004, and a significant trend at stage 2, *p* = 0.060.

In the autonomous driving mode, regardless of the complexity of the call content, the pupil diameter of the driver's left eye in the first stage is significantly different from that in the second stage (*p* = 0.037).

In manual driving mode, regardless of the complexity of the call content, the pupil diameter of the driver's left eye is significantly different in the first stage and the second stage (p = 0.000), the stage 1 and the stage 3 were significant (*p* = 0.000), and the stage 1 and the stage 4 were significant (*p* = 0.000), the stage 1 and the stage 5 were significant (*p* = 0.000), the stage 1 and the stage 6 were significant (*p* = 0.000). The stage 2 and the stage 5 were significant (*p* = 0.002), the stage 2 and the stage 6 were significant (*p* = 0.002), the stage 3 and the stage 5 were significant (*p* = 0.005), the stage 3 and the stage 6 were significant (*p* = 0.007), the stage 4 and the stage 5 were significant (*p* = 0.003), the stage 4 and the stage 6 were significant (*p* = 0.010).

**Figure 4:**
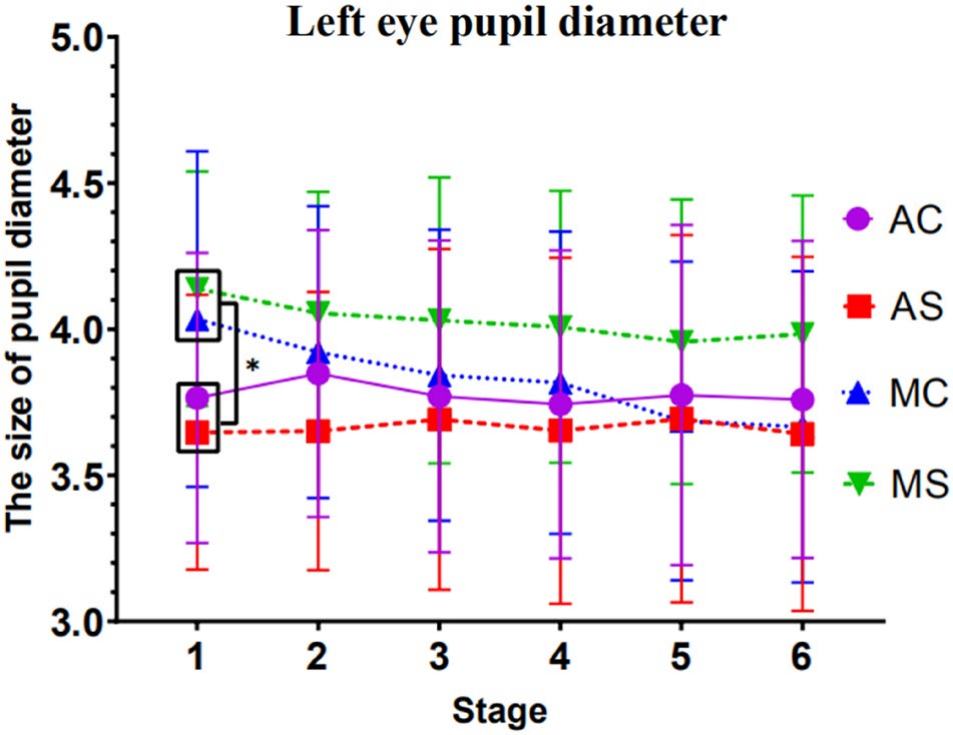
Trend in pupil diameter in the left eye (Note: Vertical bars represent standard errors, *p < 0.05).

Apart from this, no other factors or interactions reached significance. See Fig. 4 for detailed trends.

#### Results of right eye pupil diameter analysis

Results showed a significant main effect of driving mode *F*(1, 54) = 4.050, *p* = 0.049, *ηp*^*2*^ = 0.070 and a significant interaction between stage and driving mode, *F*(1, 54) = 7.665, *p* = 0.008, *ηp*^*2*^ = 0.124. Simple effect tests demonstrated that different driving modes appeared to be significantly different at stage 1 (*p* = 0.001) and stage 2 (*p* = 0.016). The three-way interaction of stage, driving mode, and call content complexity was significant *F*(1, 54) = 5.290, *p* = 0.025, *ηp*^*2*^ = 0.089. Simple effect tests demonstrated that driver pupil diameter size varied significantly with driving mode for simple call content in stage 1 (*p* = 0.005) and stage 2 (*p* = 0.030).

The size of the driver's pupil diameter varied significantly (*p* = 0.050) in the first stage as the driving mode varied while the driver was making complex calls.

A simple effect test for stage showed that in the case of manual driving with a simple call, the pupil diameter of the right eye was significantly different between the stage 1 and the stage 2 (*p* = 0.000), the stage 1 and stage 3 were significant (*p* = 0.001), the stage 1 and the stage 4 were significant (*p* = 0.008), the stage 1 and the stage 5 have a significant difference (*p* = 0.006), and the stage 1 and the stage 6 have a significant difference (*p* = 0.032).

In the case of manual driving with complex calls, the pupil diameter of the right eye was significantly different between the stage 1 and the stage 2 (*p* = 0.018), the stage 1 and the stage 3 were significant (*p* = 0.011), and the stage 1 and the stage 4 had a significant difference (*p* = 0.008), the stage 1 and the stage 5 were significant (*p* = 0.000), the stage 1 and the stage 6 were significant (*p* = 0.000). The stage 2 was significantly different from the stage 5 (*p* = 0.002), the stage 2 and the stage 6 were significant (*p* = 0.003). The stage 3 was significantly different from the stage 5 (*p* = 0.004), the stage 3 and the stage 6 were significant (*p* = 0.002), the stage 4 and the stage 5 were significant (*p* = 0.005), and the stage 4 and the stage 6 were significant (*p* = 0.002). Detailed trends are shown in Fig. 11.

**Figure 5:**
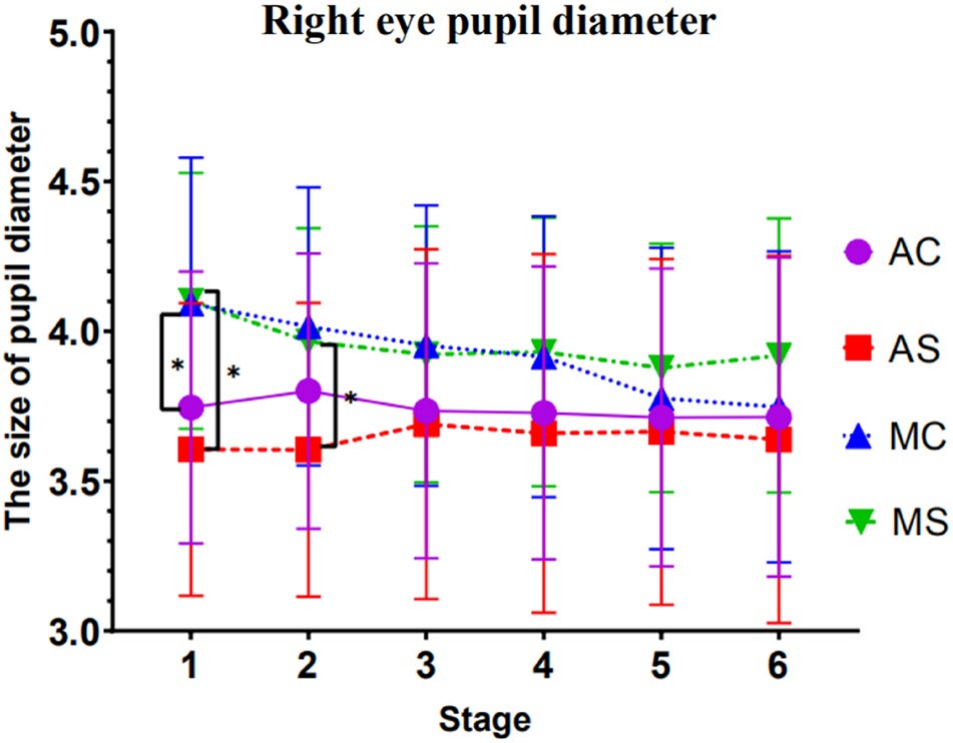
Trend in pupil diameter in the right eye (Note: Vertical bars represent standard errors, *p < 0.05).

Apart from this, no other factors or interactions reached significance. See Fig. 5 for detailed trends.

### Results of power analysis of each brain region in the EEG alpha wave band

**Figure 6:**
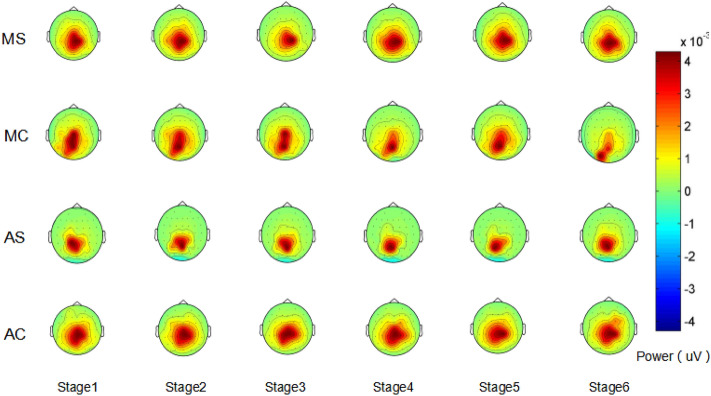
EEG alpha wave band brain topography.

The single-subject EEG spectra were averaged across subjects in each group in order to obtain the group-level EEG spectra. The alpha (8–13 Hz) power topographies were displayed as four groups and six stages (Fig. 6).

Repeated-measures ANOVA tests were conducted with driving pattern and call content complexity as between-subject variables, measurement phase as within-subject variables, and alpha wave each brain region EEG power values apart as dependent variables, with Greenhouse–Geisser correction for p-values that did not satisfy the spherical hypothesis variables. See Table 3 for the significance of factors.

**Table 3:** A summary table of the ANOVA significance of EEG (*p < 0.05).

Variables	Prefrontal lobe	Frontal lobe	Occipital lobe	Parietal lobe	Temporal lobe
Stage	0.073	0.351	0.398	0.384	0.209
Driving mode	0.763	0.447	0.039*	0.058	0.042*
Complexity	0.012*	0.721	0.672	0.028*	0.545
Driving mode*complexity	0.423	0.798	0.811	0.732	0.574
Stage*driving mode	0.013*	0.539	0.308	0.318	0.683
Stage*complexity	0.012*	0.013*	0.143	0.857	0.465
Stage*driving mode*complexity	0.010*	0.010*	0.024*	0.979	0.540

#### EEG prefrontal area power analysis results

Results showed a significant main effect of call content complexity *F*(1, 47) = 6.782, *p* = 0.012, *ηp*^*2*^ = 0.126, a significant interaction of stage with call content complexity, *F*(1, 47) = 6.836, *p* = 0.012, *ηp*^*2*^ = 0.127, and a significant interaction of stage with driving mode, *F*(1, 47) = 6.772, *p* = 0.013, *ηp*^*2*^ = 0.126). The interaction between stage, call content complexity, and driving mode was significant, *F*(1, 47) = 7.194, *p* = 0.010, *ηp*^*2*^ = 0.133.

A simple effect test of the stage showed that in the case of simple calls in autonomous driving, the EEG power values of the stage 1 and stage 2 were significant (*p* = 0.017), the stage 1 and stage 3 were significant (*p* = 0.016), the stage 1 and stage 6 were significant (*p* = 0.024) . In the case of autonomous driving complex calls, the stage 1 and stage 3 are significant (*p* = 0.039), and the stage 2 and the stage 3 are significant (*p* = 0.026). In the case of manual driving with simple calls, the stage 2 and stage 6 were significant (*p* = 0.012), and the stage 3 and stage 6 were significant (*p* = 0.009). In the case of manual driving and complex calls, there are no stage significant results.

A simple effect test of the complexity of the call content showed that in the automatic driving mode, with the different complexity of the call content, the EEG power value of the driver's alpha wave prefrontal cortex was significantly different in the first stage (*p* = 0.022).

In the manual driving mode, with the complexity of the call content, the EEG power values of the drivers were significantly different in the second stage (*p* = 0.000), the third stage (*p* = 0.005) and the fifth stage (*p* = 0.040).

A simple effect test of driving mode showed that in the case of simple call content, the EEG power value of drivers differed significantly in the second stage with different driving modes (*p* = 0.007). When conducting complex calls, there was no significant in the driver's EEG power value with different driving modes.

**Figure 7:**
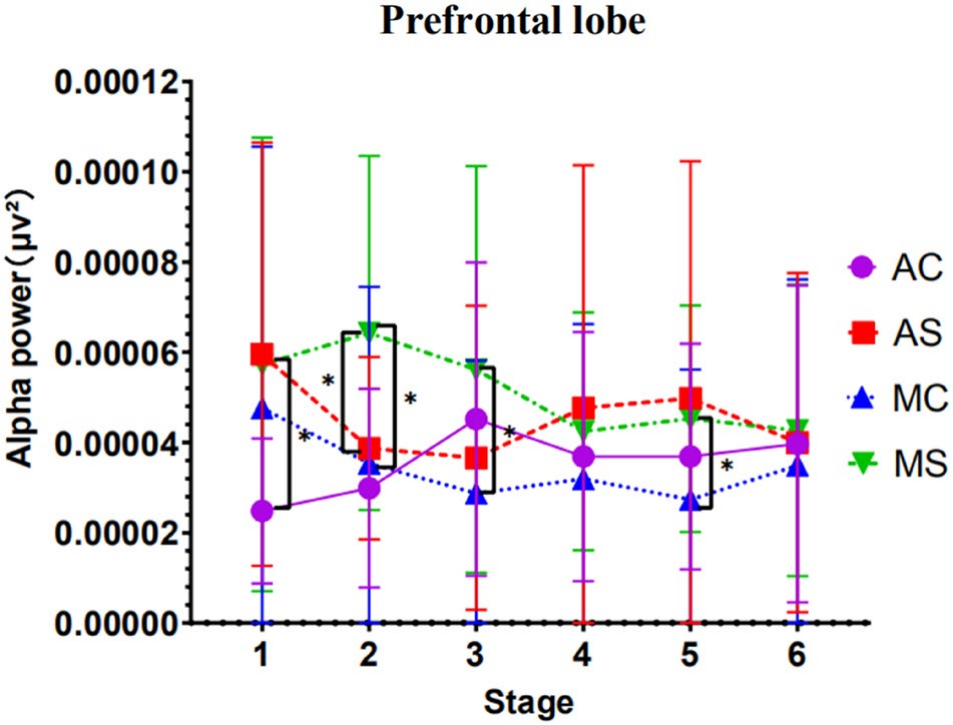
EEG alpha wave power in the prefrontal region (Note: Vertical bars represent standard errors, *p < 0.05).

Apart from this, no other factors or interactions reached significance. See Fig. 7 for detailed trends.

#### Results of power analysis of EEG frontal areas

Results showed that a significant interaction of stage with call content complexity, *F*(1, 47) = 3.509, *p* = 0.013, *ηp*^*2*^ = 0.069, and the three-way interaction of stage, driving mode, and call content complexity was significant *F*(1, 47) = 3.699,* p* = 0.010, *ηp*^*2*^ = 0.073. A simple effect test of the stage showed that in the case of simple calls in autonomous driving, the EEG power values of the stage 1 and stage 2 were significant (*p* = 0.011), the stage 1 and stage 3 were significant (*p* = 0.001), the stage 1 and stage 5 were significant (*p* = 0.009), the stage 1 and stage 6 were significant (*p* = 0.025), the stage 3 and stage 4 were significant (*p* = 0.010).

In the case of autonomous driving complex calls, the stage 1 and stage 3 were significant (*p* = 0.016), the stage 1 and the stage 6 were significant (*p* = 0.003), the stage 2 and the stage 6 were significant (*p* = 0.003), the stage 4 and the stage 6 were significant (*p* = 0.017), the stage 5 and the stage 6 were significant (*p* = 0.002).

In the case of manual driving with simple calls, the stage 1 and stage 3 were significant (*p* = 0.036), the stage 1 and stage 4 were significant (*p* = 0.006), the stage 3 and the stage 5 are significant (*p* = 0.007), the stage 4 and the stage 5 are significant (*p* = 0.045). In the case of manual driving and complex calls, there are no stage significant results.

A simple effect test of the complexity of the call content showed that in the automatic driving mode, with the different complexity of the call content, the EEG power value of the driver's alpha wave frontal lobe area was significantly different in the first stage (*p* = 0.015).

In the manual driving mode, with the different complexity of the call content, the driver's brain power value was significantly different in the fifth stage (*p* = 0.023).

A simple effect test of driving mode showed that in the case of complex call content, with the different driving modes, the driver's EEG power value was significantly different in the sixth stage (*p* = 0.027). When conducting a simple call, there was no significant in the EEG power value at each stage with different driving modes.

**Figure 8:**
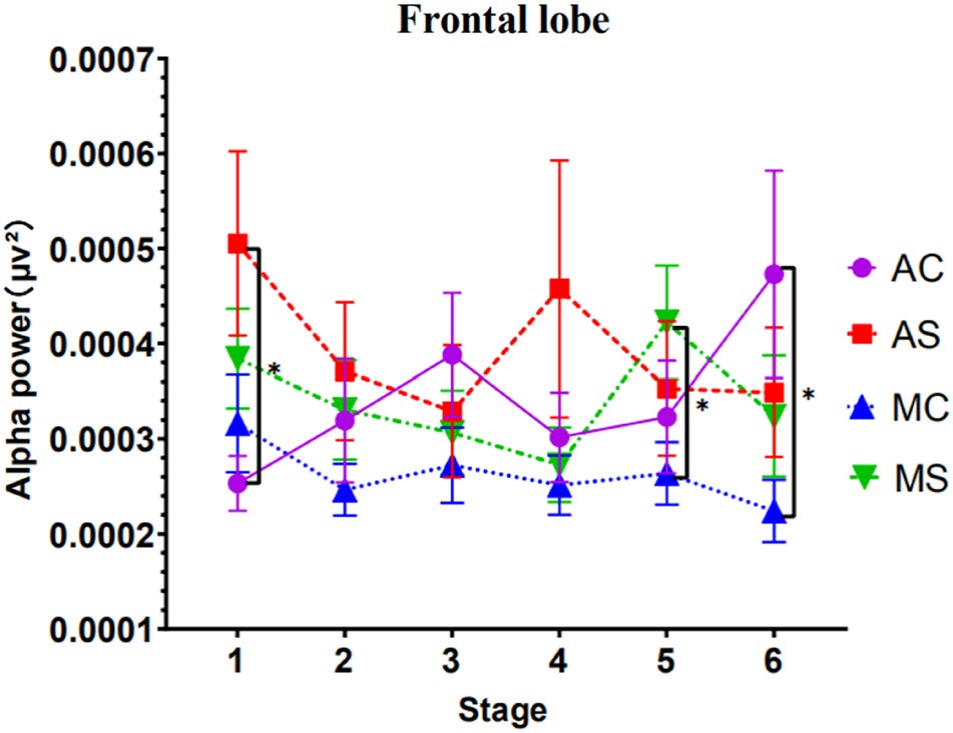
EEG frontal area alpha wave power (Note: Vertical bars represent standard errors, *p < 0.05).

Apart from this, no other factors or interactions reached significance. See Fig. 8 for detailed trends.

#### Results of power analysis of EEG occipital area

Results showed a significant main effect of driving mode *F*(1, 47) = 4.528, *p* = 0.039, *ηp*^*2*^ = 0.088, and a significant three-factor interaction of stage, driving mode, and call content complexity *F*(1, 47) = 5.409, *p* = 0.024, *ηp*^*2*^ = 0.103.

A simple effect test of the stage shows that in the case of automatic driving simple call, the EEG power value of the stage 1 and the stage 6 were significant (*p* = 0.012), the stage 2 and the stage 6 were significant (*p* = 0.027), the stage 4 and the stage 6 were significant (*p* = 0.034). In the complex call situation of autonomous driving, the EEG power values of the stage 1 and the stage 3 were significant (*p* = 0.024). Apart from this, no other factors or interactions reached significance.

A simple effect test of the complexity of the call content shows that no matter which driving mode, there is no significant difference in the EEG power value of the driver with the complexity of the call content.

A simple effect test of driving mode showed that in the case of simple calls content, the EEG power values of drivers was significant difference in the first stage with different driving modes (*p* = 0.023). In the case of complex call content, with different driving modes, the driver's EEG power value was significant difference in the third stage (*p* = 0.048) and the fifth stage (*p* = 0.027).

**Figure 9:**
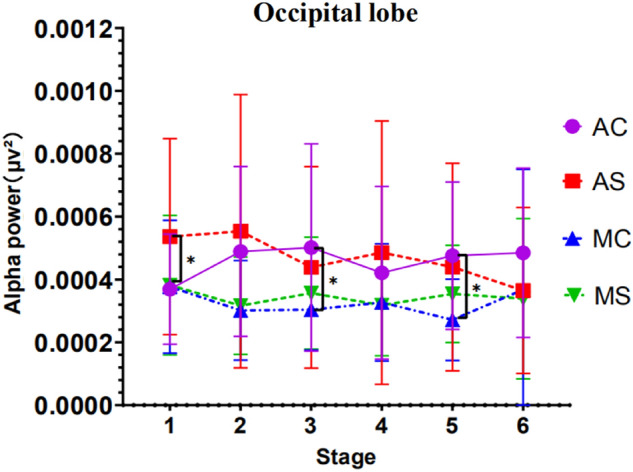
EEG alpha wave power in the occipital region (Note: Vertical bars represent standard errors, *p < 0.05).

Apart from this, no other factors or interactions reached significance. See Fig. 9 for detailed trends.

#### Results of power analysis of the parietal region of the EEG

**Figure 10:**
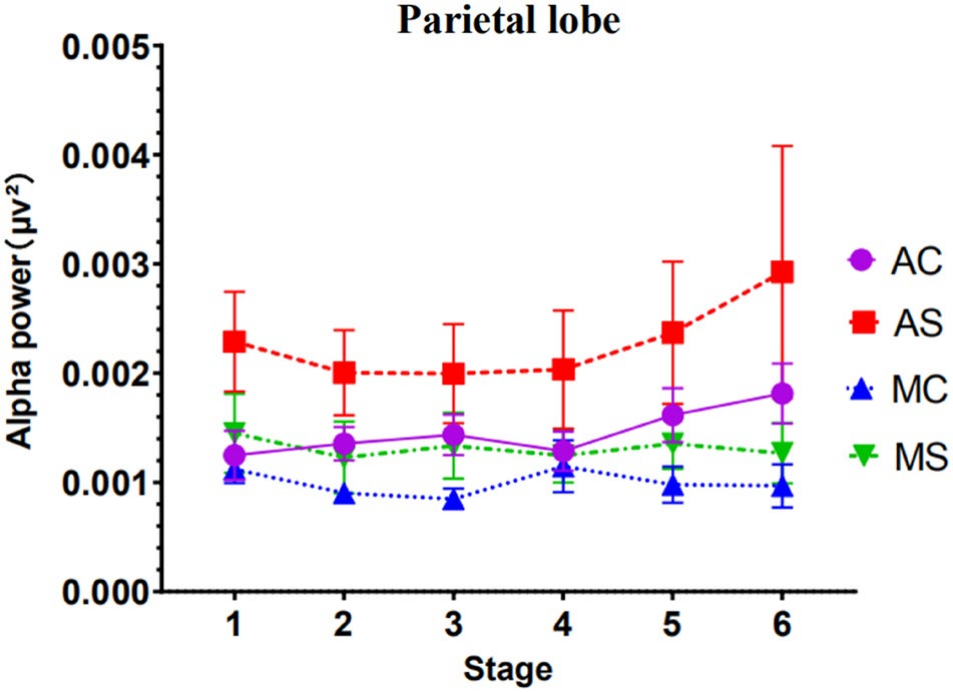
EEG parietal region alpha wave power (Note: Vertical bars represent standard errors, *p < 0.05).

Results showed that the main effect of call content complexity was significant *F*(1, 47) = 5.746, *p* = 0.028, *ηp*^*2*^ = 0.074. Apart from this, no other factors or interactions reached significance. See Fig. 10 for detailed trends.

#### Results of EEG temporal lobe area power analysis

Results showed a significant driving mode main effect *F*(1, 47) = 5.732, *p* = 0.042, *ηp*^*2*^ = 0.081.

**Figure 11:**
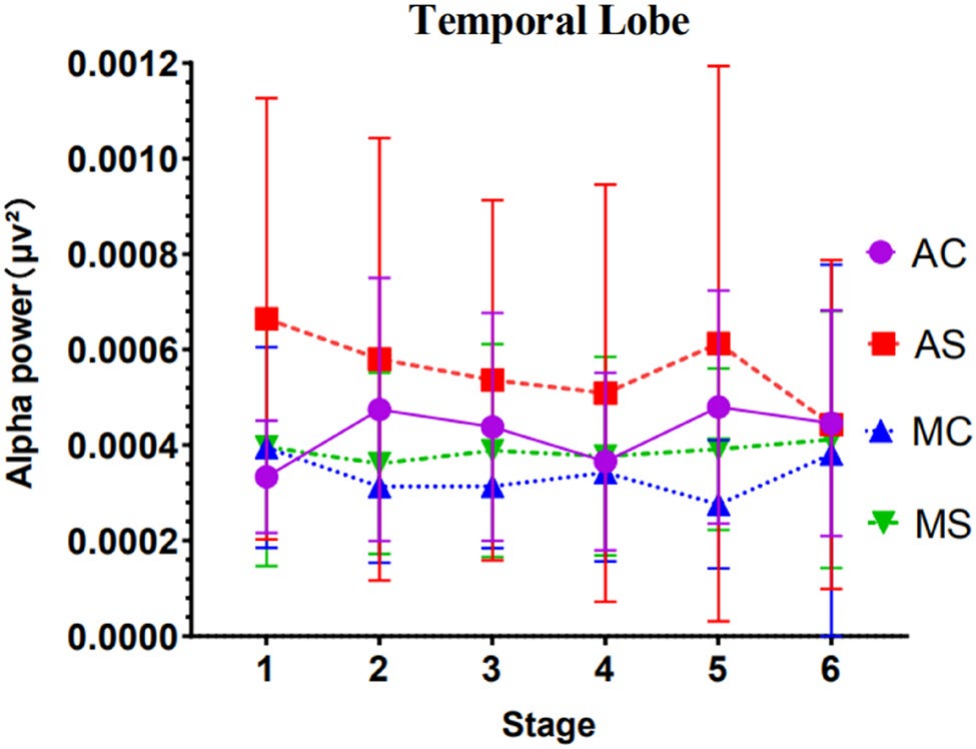
EEG alpha wave power in the temporal lobe region (Note: Vertical bars represent standard errors, *p < 0.05).

Apart from this, no other factors or interactions reached significance. See Fig. 11 for detailed trends.

## Discussion

The analysis of the detection response task shows that when the driver is talking on the mobile phone in the initial stage of driving (about 10 min), the reaction time in the manual driving group is significantly longer than that in the automated driving group, while the accuracy of detection response task in the manual driving group is significantly lower than that in the automated driving group. As the driving time increased, the automated driving group’s reaction time of detection response gradually became longer, consistent with that of the manual driving group. Moreover, it tends to surpass the manual driving group after 60 min. This shows that although the automatic driving system alleviates the excessive cognitive load caused by mobile phone calls at the initial stage of driving, as the driving time increases, the negative impact of mobile phone calls on drivers still exists, and it may become more serious, thus affecting driving safety. In the manual driving mode, regardless of the complexity of the mobile phone call, the driver's reaction time and accuracy in the fourth and fifth stages are significantly different from those in the first stage. From the trend in Figs. 2 and 3, it shows that the driver's reaction time is significantly faster, and the accuracy rate is improved during the driving time of 10–50 min. This may be due to the practice effect produced by the participants constantly making phone calls every ten minutes during the experiment.

About pupil diameter data, under simple conversation conditions, we found that at the initial stage of driving (within10–20 min), the pupil diameter of the driver in the automatic driving group was significantly smaller than that in the manual driving group. However, with the increase in driving time, there is no significant difference in pupil diameter between the automatic and the manual driving groups. This follows the trend of reaction time of detection response task. At the same time, we also tested the results of the simple effect between the pupil diameters of the left and right eyes, and found that under the condition of manual driving and complex call content, the pupil diameters of the participants in each stage were significantly different from those in the sixth stage. The second, third, and fourth stages were both significantly different from the pupil diameter of the fifth stage. As the driving time increases, complex mobile phone calls cause the pupil diameter of the driver to shrink gradually. This shows that when the driving time was 50 to 60 min, the pupil diameter of the driver had a significant shrinking trend. The eye movement research in the reading field shows that the increase of mental workload will induce the pupil diameter to expand on one hand. At the same time, with the extension of working time, fatigue factors will activate the parasympathetic nerve and inhibit the sympathetic nerve from making the sphincter contract, thus making the pupil shrink^38^. Therefore, the changing trend of pupil diameter indicates that in manual driving mode, when the driving time is about 50–60 min, the mental workload and fatigue caused by call content will have an antagonistic effect and jointly restrict the contraction of pupil diameter.

The driver's brain regions cooperate to complete the driving task together during driving. The distraction effect is due primarily to the conversion of brain resources^16^. Furthermore, our research also found that cell phone calls had different adverse effects on each brain regions.

Firstly, in the prefrontal area, we found that when the driving time was 10 min in the automatic driving mode, the alpha wave power value in the prefrontal area of the complex conversation content group was significantly lower than that of the simple conversation content group. When the driving time is 20–30 min in manual driving mode, the alpha power value in the prefrontal area of the complex call content group is significantly lower than that of the simple call content group. However, with the increase in driving time, the difference gradually decreases, with no significant difference between them. When the driving time is 60 min, the driver's mental workload level tends to be consistent regardless of the driving mode and the complicated conversation content. This shows that, in the same driving mode, the mental workload caused by the complexity of call content is significantly different only in the early driving stage. The mental workload caused by complex conversation is higher, and the mental workload caused by simple conversation is lower. With the increase in driving time, the difference gradually disappears and tends to be consistent.

Also, in the prefrontal area, an important finding is that at 20 min of driving, the alpha power values in the prefrontal areas of the simple talk content group were significantly lower than those of the simple talk group in the manual driving group. And in the case of simple talk in automatic driving, the driver’s first stage EEG power values of the prefrontal area and the frontal area were significantly different from the second and third stages, their EEG power values are within a specific driving stage (10–20 min, 10–30 min) decreased rapidly, and its level of mental workload quickly increased during this stage.

The prefrontal cortex has been thought to play an essential role in cognitive control, i.e., the coordination of thoughts and actions according to internal goals. Cognitive control stems from the active maintenance of prefrontal cortex activity patterns that represent goals and the means to achieve those goals^23^. Thus, the results of the EEG data suggest that the mental workload induced by the content of a simple mobile phone call is higher in the automatic driving mode for about 10–20 min of driving time than in the manual driving state during the same period, occupying more brain resources in the driver's prefrontal area and leading to a reduction in his or her cognitive control. At the same time, the trends in the detection response task data during this stage were consistent with the EEG data. The trend in Fig. 2 shows that at a driving duration of 10 min, the automatic driving simple talk content group had faster response times than the manual driving simple talk content group. When driving for 20 min, the autopilot simplex content group's response times slowed rapidly and converged with the manual simple content group. Although the trend in Fig. 3 indicates that the accuracy of autopilot simple content group was higher than the manual group during this period, the EEG data shows that the overall driver load was still high during this period, as their cognitive control was reduced and the excessive attentional resources were disruptive to the driver's reaction time to detect peripheral signals, resulting in progressively slower reaction times.

In the frontal lobe area, we found that the driving time in the automated driving mode was about 10 min, the alpha wave power value in the frontal lobe area of the complex call content group was significantly lower than that of the simple call content group. This is consistent with the previously reported data trend in the prefrontal region. The difference in the mental workload produced by different complexity of the call content is only significant in the early driving period.

Also, previous studies have found that drivers performing interactive cognitive tasks during prolonged driving appear to improve alertness and driving performance^39^. And through the trend of EEG data in the frontal lobe and occipital lobe, we found that in the autopilot mode, drivers in the complex mobile phone talk content group had significantly higher alpha wave power values in the occipital region than the manual mode complex talk content group for the same period (30–50 min), and that the complex mobile phone talk content group had significantly higher alpha wave power values in the frontal region than the manual mode complex talk content group for the same period (60 min). Since power changes in frontal areas mainly reflect the degree of activation of intrinsic neurons when individuals allocate their attention to different task stimuli^22^. However, parietal circuits, the prefrontal cortex, and corticolimbic structures were shown to be involved in the distribution of individual directed attention networks together with the medial pulvinar nucleus (Bakeydier and Mauguiere, 1985). This nucleus projects and receives visual input from the occipital cortex and the superior colliculus, forming an essential link with the hippocampus for further memory processing^40^. This suggests that the effects of mobile phone conversations on activation of the occipital cortex and the frontal cortex are shared. At the same time, frontal areas are involved in impulse control, judgment, language production, working memory, motor function, and problem-solving^21^. This shows that when drivers engage in complex call content, even when on autopilot, brain functions such as problem-solving, judgment , and impulse control are impaired as the duration of driving increases. This study also refines the findings of Saxby et al.^4^ by exploring the complexity of mobile phone calls, and finds that even complex calls, in monotonous prolonged autopilot mode, are not a safe way to reduce fatigue and increase alertness, but instead can be detrimental to driver function in various brain regions.

In the frontal lobe area, we also found that there was a significant difference between the driver's EEG power value in the third stage and the fourth stage EEG power value in the case of simple calls in automatic driving. From the trend in Fig. 8, the EEG power value of the fourth stage is significantly higher than that of the third stage, which indicates that the level of the driver's mental workload drops dramatically when the driver makes a simple mobile phone call during the driving time of 30–40 min in the automatic driving mode. The research of Zhang et al.^35^ shows that in the monotonous driving environment of autonomous driving, the driving time of about 40 min is a critical period for the generation of passive fatigue of the driver, which is manifested in the rapid reduction of the driver's mental workload level during this period. This indicates that simple mobile phone calls cannot be used as an effective strategy to improve driver vigilance during this period, nor can they alleviate the driver's passive fatigue level. Meanwhile, Naujoks et al.^41^ found that in autonomous driving mode, drivers with lower mental workload participated more frequently in non-driving-related tasks. This appears to be a behavioral feature of drivers regulating their mental workload. This also explains why, in the trend in Fig. 8, we observed that between the fourth and fifth stages of autonomous driving mode, the driver's mental workload for simple mobile phone calls increased rapidly again, which may be the driver's use mobile phone calls to regulate the stability of their mental workload.

Although no significant interaction results between related variables were found in the data of EEG power values in the parietal lobe and temporal lobe, the changing trend of EEG power values in the two brain regions is worthy of reference. When the driving time is 60 min, the trend of temporal lobe data shows that the driver's mental workload level tends to be consistent regardless of the driving mode and the complexity of the conversation. This is consistent with the trend of data results previously reported. Under the condition of simply call in the automated driving mode, the data of the prefrontal, frontal and occipital areas all found that the EEG power value of the driver in the first stage was significantly different from the sixth stage. The EEG power value was significantly lower than the EEG power value of the first stage. The trend of EEG power values in the temporal area is also consistent with the previous trend. The above trends all indicate that when the driver makes simple mobile phone calls in the automated driving mode, as the driving time increases, the mental workload of the driver is also increasing and accumulating. When the driving time is about 60 min, the mental workload level of the driver is significantly higher than that at the beginning of the driving stage (about 10 min). At the same time, the parietal lobe is usually closely related to acquiring and integrating sensory information during driving. The temporal lobe is mainly responsible for processing auditory information during driving, such as car whistles^42^. Therefore, in the study of driving distraction, data trends in the parietal and temporal EEG bands other than alpha waves may be more effective for detecting other distracting behaviors, and further research is needed.

Almahasneh’s^19^ study found that the most affected brain region during distracted driving was the right frontal cortex. The present study builds on this finding by examining the complexity of drivers' secondary tasks. The most significant difference in the detection of distracted driving was found in the prefrontal areas of the frontal lobe when the complexity of the secondary task was varied, suggesting that mobile phone conversations impair cognitive control in the prefrontal areas of the driver. Meanwhile, Lin et al.^20^ suggested that changes in the alpha band in the frontal region were associated with distracted driving. The EEG results of the present study indicate that a decrease in alpha wave power values in the prefrontal area is a valid indicator for identifying increased mental workload in drivers due to distracted mobile phone use; this complements another brain region band indicator for the detection of driving distraction^15,43,44^.

For the parietal region, alpha power values were higher in the simple call content group than in the complex call content group at stage 6. In the frontal and occipital regions, the alpha power values of the simple call content group were lower than those of the complex call content group at stage 6. A possible explanation for this is that the occipital area is very close to the parietal area, and the left and right motor areas of the frontal lobe. Because of the interconnectedness and complexity of the brain areas, brain areas located in the same cortical layer interact with each other while acting separately on the body^40^. It is also possible that at the end of the driving phase, EEG signal acquisition is affected by the driver's somatic fluctuations, resulting in variable alpha-wave brain power values during stage 6.

It should be noted that the EEG power in this study is low. It may be caused by the calculation method of power. Due to the large additional fluctuations caused by simulated driving activities, the artifact removal criteria for EEG are stricter.^45^After probabilistic mapping-based artifact detection and single-channel EEG signal removal, the signal value is not high. This is also true in previous literature, such as Siddiqui's study^46^ using channel ROC-LOC to apply the PSD method to EEG signal short-term frequency analysis to diagnose insomnia and sleep disorders in the study, the beta band power variation range is 0.0005–0.007, the value is not high. Therefore, even if the value of Alpha power is low (basically between 0.0013 and 0.0025), we still report them objectively. The size of the final absolute value does not affect the conclusion, because we are discussing the mental workload; the relative change of the power value makes more sense, and can better reflect the influence of mental workload on EEG.

As automated systems move towards higher levels (L4 ~ L5), the frequency and risk of mobile phone use by drivers remain a key safety concern. Future research needs to further explore the ecology and empirical evidence of mobile phone call content; delineate the impact of mobile phone calls on the conversion of resources in the driver's brain area at other wave frequencies; distinguish how the cognitive load from mobile phone calls and the mental workload from fatigue cross over to affect the driver in automated driving mode; and how the mental workload on the driver's mobile phone use changes in longer (more than 60 min) automated driving situations, as well as how the mental workload of mobile phone use changes in longer (more than 60 min) autonomous driving situations.

In summary, this study compares the safety implications of mobile phone distraction in L2 autonomous driving mode with the use of mobile phones in conventional driving situations; provides a rough delineation of when and for how long mobile phone conversations take place; compares the changes in EEG components in various brain regions in the driver's alpha wave band during mobile phone conversations. This provides a theoretical basis for the development of laws and regulations and policy implications for the use of mobile phones in the field of autonomous driving, where L2 autonomous driving systems require drivers to constantly monitor road hazards and be ready to take over the vehicle, and where the overload caused by mobile phone use can compromise driving safety. At the same time, mobile phone conversations are not a consistent and effective response to the problem of underload in L2 autonomous driving, as they can impair the normal functioning of the driver's brain functions, such as cognitive control, problem-solving, and judgment, while increasing the mental workload and compromising driving safety.

## Conclusion

During the initial phase of driving (10–20 min), the mental workload induced by the content of a simple mobile phone call is higher in L2 automated driving mode than in manual driving during the same stage, occupying more brain resources in the driver's prefrontal area, leading to a reduction in his cognitive control, and making the driver slower to respond to peripheral visual detection signals.

The activation of the driver's occipital area in the complex call content group was lower in the L2 automated driving mode compared to the manual driving mode during the same stage (30–50 min), and the activation of the frontal area in the complex call content group was lower compared to the manual driving mode during the same stage (60 min), indicating that complex mobile phone calls, even in the L2 automated driving state, caused a decline in the driver's problem-solving, judgment, and impulse control, among other brain functions.

The changing trend of pupil diameter indicates that in manual driving mode, when the driving time is about 50–60 min, the mental workload and fatigue caused by call content will have an antagonistic effect and jointly restrict the contraction of pupil diameter.

EEG and detection response task indicators together indicate that regardless of the level of complexity of mobile phone calls made by the driver, the level of mental workload tends to be the same for drivers in manual and L2 automated driving modes as the duration of driving increases, suggesting that mobile phone calls in L2 automatic driving mode also disrupt the driver's cognitive resource balance, leading to a reduction in cognitive control and impairing driving safety.

## Supplementary Information

Supplementary Information.

## Data Availability

All data generated or analysed during this study are included in this published article [and its supplementary information files].
